# Effects of Hydrolysis Reaction and Abrasive Drag Force Accelerator on Enhancing Si-Wafer Polishing Rate and Improving Si-Wafer Surface Roughness

**DOI:** 10.3390/nano15161248

**Published:** 2025-08-14

**Authors:** Min-Uk Jeon, Pil-Su Kim, Man-Hyup Han, Se-Hui Lee, Hye-Min Lee, Su-Bin Kim, Jin-Hyung Park, Kyoo-Chul Cho, Jinsub Park, Jea-Gun Park

**Affiliations:** 1 Department of Electronic Engineering, Hanyang University, Seoul 04763, Republic of Korea; mujeon1214@gmail.com (M.-U.J.); eiop77455@gmail.com (S.-H.L.); hm0951911@gmail.com (H.-M.L.); emptybin.kim@gmail.com (S.-B.K.); jinsubpark@hanyang.ac.kr (J.P.); 2 Department of Nanoscale Semiconductor Engineering, Hanyang University, Seoul 04763, Republic of Korea; psk6208@naver.com (P.-S.K.); aksguq06@naver.com (M.-H.H.); 3 ENF Technology Co., Ltd., Yongin 17084, Republic of Korea; parkjinhyung@gmail.com; 4 Advanced Semiconductor Materials & Device Development Center, Hanyang University, Seoul 04763, Republic of Korea; kccho12@naver.com

**Keywords:** silicon CMP, colloidal silica slurry, High-Bandwidth Memory (HBM), triammonium phosphate (TAP), hydroxyethyl cellulose (HEC)

## Abstract

To satisfy the superior surface quality requirements in the fabrication of HBM (High-Bandwidth Memory) and 3D NAND Flash Memory, high-efficiency Si chemical mechanical planarization (CMP) is essential. In this study, a colloidal silica abrasive-based Si-wafer CMP slurry was developed to simultaneously achieve a high polishing rate (≥10 nm/min) and low surface roughness (≤0.2 nm) without inducing CMP-induced scratches. The proposed Si-wafer CMP slurry incorporates two functional components: triammonium phosphate (TAP) as a hydrolysis reaction accelerator and hydroxyethyl cellulose (HEC) as an abrasive drag force accelerator. The polishing rate enhancement mechanism of TAP was analyzed by monitoring the OH^−^ mol concentration, surface adsorption behavior, and XPS spectra. The results showed that increasing the TAP concentration raised the OH^−^ mol concentration and converted Si–Si and Si–O–Si bonds to Si–OH via a hydrolysis reaction, thereby increasing the polishing rate. However, excessive hydrolysis also led to increased surface roughness. On the other hand, HEC influenced slurry viscosity, abrasive dispersibility, and drag force. At low HEC concentrations, increased abrasive drag force improved the polishing rate. At high concentrations, however, HEC formed a hindrance layer on the Si surface via hydrogen bonding and condensation reactions, reducing the effective contact area of abrasives and thus decreasing the polishing rate. By optimizing the concentrations of TAP (0.0037 wt%) and HEC (≤0.0024 wt%), the proposed slurry formulation achieved high-performance Si-wafer CMP, satisfying both surface roughness and polishing rate targets required for advanced memory packaging applications.

## 1. Introduction

The recent advancement of the AI software industry has been accompanied by the development of high-performance AI accelerators. AI accelerators are packaged systems where multiple GPUs are connected with High-Bandwidth Memory (HBM) [1,2,3]. To increase bandwidth, HBM enhances the speed of DRAM and increases the data processing input/output units [4,5,6,7,8]. Moreover, in order to run software applying a Large Language Model (LLM), large amounts of data must be processed in a short time, which requires an increase in HBM capacity [9]. Currently, HBM is manufactured by vertically stacking 8 layers of DRAM, and it is known that 12 or 16 layers of DRAM will be vertically stacked in the near future [10,11,12,13]. To produce HBM, the DRAM-fabricated wafer should be thinned down to several tens of micrometers and undergo TSV formation [14,15,16]. The 775 μm thick Si-wafer is thinned through grinding to several tens of micrometers, and after thinning, it must achieve less than 1 μm of Si Total Thickness Variation (TTV) and less than 1 nm of Si surface roughness by dry etching and Si surface CMP using a soft pad (i.e., 71 shore A) to remove grinding damage [17,18,19,20,21,22]. In addition, in recent 3-dimensional (D) NAND Flash Memory, memory cell wafers stacked vertically are bonded with wafers containing periphery circuits through silicon via (TSV), and 775 μm thick wafers are thinned to several tens of micrometers through grinding, dry etching, and Si polishing using a soft pad [23,24]. Unlike a conventional thinning via Si polishing using a hard pad (i.e., 95 shore A) showing an extremely high Si polishing rate [25], a recent thinning method was applied by dry etching and the Si-wafer CMP using a soft pad showing a proper Si polishing rate (i.e., ≥10 nm/min) and extremely low Si surface roughness after CMP (i.e., ≤0.2 nm). Therefore, Si-wafer CMP using a soft pad has become an essential process thinning the wafers of HBM and 3D NAND Flash Memory. Particularly, the Si-wafer CMP slurry designed with the reported hydrolysis reaction accelerator (i.e., ethylenediamine: EDA) could not achieve a sufficient Si polishing rate (i.e., 2.2 nm/min) when the CMP polisher used a soft pad (i.e., 71 shore A), as shown in Figure S1.

In this study, a Si-wafer CMP slurry was designed to achieve a Si polishing rate above 10 nm/min, extremely lower surface roughness below 0.2 nm after the Si-wafer CMP using a soft pad, and less CMP-induced scratches. The Si-wafer CMP slurry was designed with colloidal silica abrasives, a hydrolysis reaction accelerator (i.e., triammonium phosphate, TAP: (NH_4_)_3_PO_4_) [26,27,28,29,30,31,32,33], an abrasive drag force accelerator (i.e., hydroxyethyl ether: HEC) [34,35,36], a pH titrant, and DIW that enhances the Si polishing rate, minimizes Si surface roughness, and reduces CMP-induced scratches. First, the Si polishing rate and the Si surface roughness were evaluated depending on the hydrolysis reaction accelerator (i.e., TAP) concentration [26,27,28,29,30,31,32,33]. To understand the mechanism by which the polishing rate increases with the TAP concentration, the adsorption degree of CMP slurry on the Si-wafer surface and the chemical composition of the polished Si surface were analyzed using XPS [37]. Second, the Si polishing rate and the Si surface roughness were estimated depending on abrasive drag force accelerator (i.e., HEC) concentration [34,35,36]. Note that the designed Si-wafer CMP slurry mixed simultaneously via the hydrolysis reaction and abrasive drag force accelerator after the addition of the hydrolysis reaction accelerator itself in the Si-wafer CMP slurry was insufficient to achieve a proper Si polishing rate. To understand the mechanism by which the Si polishing rate changes with an increasing HEC concentration in the Si-wafer CMP slurry, the slurry viscosity, adsorption degree on the polished Si surface, and chemical composition of the polished Si surface using XPS were observed [37].

## 2. Materials and Methods

### 2.1. Materials

The CMP process was carried out using (100)-oriented silicon wafers. In the experiments, colloidal silica abrasives with an average diameter of 100 nm dispersed in deionized water (SS-SOL 100, S-CHEMTECH Co., Ltd., Ansan, Republic of Korea) were used. The Si-wafer CMP slurry consisted of 0.22 wt% colloidal silica abrasives, triammonium phosphate trihydrate (TAP: Aladdin, analytical reagent, 98.0%, powder) as a Si polishing rate accelerator, and hydroxyethyl cellulose (HEC: Polysciences, ≤100%, powder) as a second polishing rate accelerator. Note that TAP accelerates significantly a hydrolysis reaction between hydroxyl ions and dangling bonded Si atoms on the Si surface via generating OH ions during Si-wafer CMP. HEC enhances notably the abrasive drag force in the slurry via increasing the viscosity of the slurry during Si-wafer CMP. The pH of the colloidal silica abrasive itself was 9.7, and the slurry was prepared by stirring at 300 rpm for 30 min at room temperature (25 °C). The final slurry pH was adjusted to 9.7 using NH_4_OH. This pH was selected to ensure sufficient OH^−^ ion concentration to promote Si surface hydrolysis while maintaining stable dispersion (zeta potential: > −40 mV) of colloidal silica abrasives. Previous studies have demonstrated that colloidal silica slurries exhibit optimal dispersion and polishing characteristics in the alkaline pH range of 9.5–10.5 [25].

### 2.2. CMP Conditions

**Figure 1:**
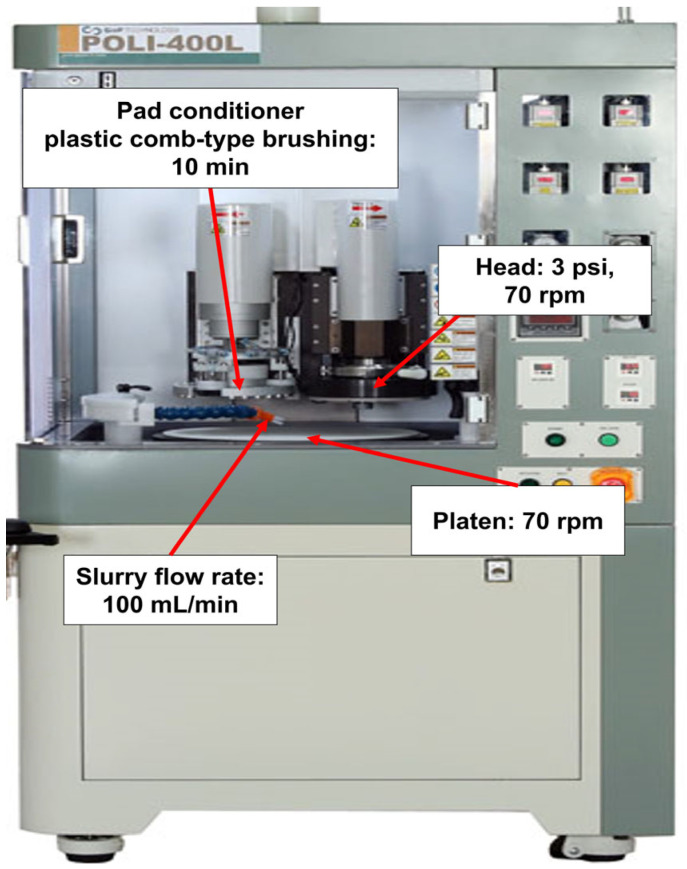
A photograph of the CMP polisher (POLI-400).

**Figure 2:**
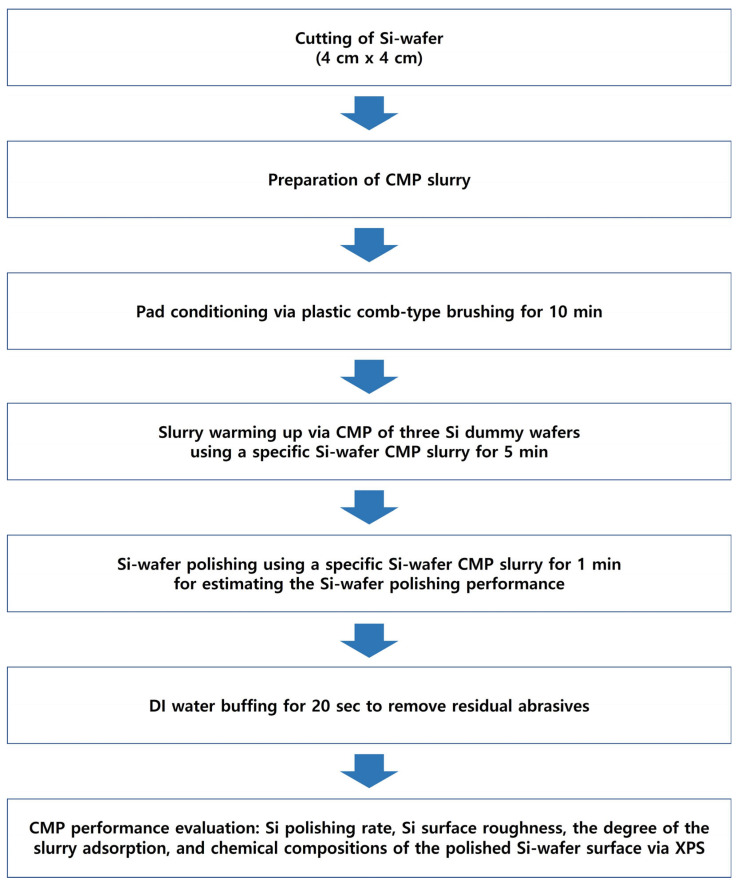
Schematic diagram of the experimental procedure for Si-wafer CMP.

The 12-inch (100)-oriented Si-wafers were diced into 4 cm × 4 cm square samples for CMP evaluation. The polishing was performed using a CMP polisher (POLI-400, G&P Tech. Inc., Busan, Republic of Korea) equipped with a patterned CMP pad (CM4307NX, FUJIBO Ehime Co., Ltd., Tokyo, Japan), as shown in Figure 1. Prior to CMP, the pad was conditioned for 10 min, and three dummy wafers were polished to stabilize the pad for each slurry composition, as shown in Figure 2. CMP was conducted under the following conditions: head pressure of 3 psi, head rotation speed of 70 rpm, and platen rotation speed of 70 rpm. The slurry flow rate was fixed at 100 mL/min, and each polishing process was conducted for 20 min. After polishing, wafers were rinsed by buffing in DI water for 20 s to remove residual abrasives from the surface.

### 2.3. Measurements

The secondary abrasive diameter and zeta potential of the Si-wafer CMP slurry were measured using a particle size and zeta potential analyzer (ELSZ-2000ZS, Otsuka Electronics Co., Inc., Osaka, Japan). The viscosity of the slurry was evaluated using a viscometer (DV-II+Pro, Ametek Brookfield, Middleboro, MA, USA). The odor level of the slurry was measured using a portable odor monitor (OMX-ADM, Shin-Etsu Chemical Co., Ltd., Tokyo, Japan). The Si polishing rate was calculated by measuring the weight of the wafer before and after CMP [38], using Equation (1):

(1) $${polishing\;rate\;}_{Si}=\;∆m/(\rho \cdot A\cdot t)$$

where Δ*m* is the change in the Si-wafer mass, *ρ* is the Si-wafer density (2.33 g/cm^3^), *A* is the polished surface area, and *t* is the polishing time. The surface roughness of the polished Si-wafer was measured using atomic force microscopy (AFM, Park Systems, Suwon, Republic of Korea) over a 2 μm × 2 μm scan area, with 512 pixels × 512 lines and a scan rate of 1.0 Hz. The drag force for colloidal silica abrasives in the slurry was calculated using Equation (2):

(2) $$F_{d}=3\pi \mu dv$$

which is derived from Stokes’ law. This equation describes the viscous resistance experienced by a spherical particle moving through a fluid. Therefore, applying this equation is appropriate for evaluating the drag force acting on colloidal silica abrasives during the CMP process [39]. $F_{d}$ is the drag force (N), μ is the viscosity of the slurry (cP), d is the diameter of the colloidal silica abrasive (nm), and v is the relative velocity between the slurry and the abrasive (m/s). To measure the adsorption degree on the Si-wafer surface, the wafer was immersed in the CMP slurry at a 45° angle for 1 min and then vertically dried for 1 min prior to analysis. This dipping method has been widely used in CMP to evaluate the interaction between the slurry and wafer surface [39,40]. The chemical composition of the polished Si surface was analyzed using X-ray photoelectron spectroscopy (XPS, K-Alpha^+^, Thermo Fisher Scientific Co., Inc., Waltham, MA, USA) under conditions of 12 keV and 6 mA.

## 3. Results and Discussion

### 3.1. Dependency of Si Polishing Rate and Polished Si Surface Roughness Increase on Hydrolysis Reaction Accelerator (i.e., TAP) in Si-Wafer CMP Slurry

**Figure 3:**
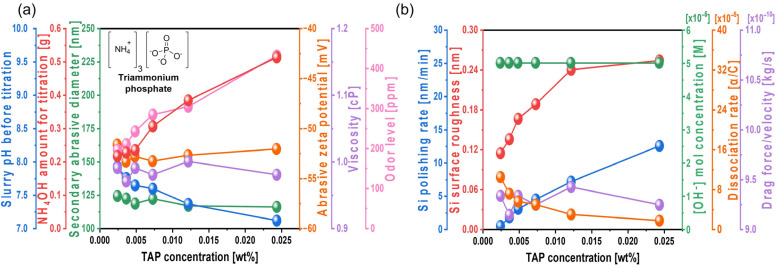
Dependency of Si-wafer CMP slurry properties and polishing performance on TAP concentration. (**a**) Slurry properties, including slurry pH before titration, NH_4_OH consumption for pH adjustment, secondary abrasive diameter, abrasive zeta potential, viscosity, and odor level, and (**b**) Si-wafer CMP performance, including polishing rate, surface roughness, hydroxide ion concentration, dissociation rate of hydroxide ion, and drag force.

To enhance the silicon polishing rate, triammonium phosphate trihydrate (TAP), which contains phosphate groups that accelerate hydrolysis reactions on the Si surface, was mixed to the Si-wafer CMP slurry containing 0.22 wt% colloidal silica abrasives at pH 9.5. As the TAP concentration increased from 0.0024 to 0.0244 wt%, the slurry pH before titration decreased from 7.92 to 7.11, and the amount of NH_4_OH required to maintain the slurry pH at 9.7 significantly increased from 0.216 (i.e., 0.246 mol) to 0.513 g (i.e., 0.586 mol), as shown in Figure 3a. This is because TAP dissociates into NH_4_^+^ and PO_4_^3−^ in aqueous solution, as shown in Equation (3). The dissociated NH_4_^+^ further ionizes into NH_3_ and H^+^, thereby acidifying the solution, as shown in Equation (4). Therefore, as the TAP concentration increases, the solution becomes more acidic, necessitating additional NH_4_OH to maintain the pH at 9.7, as shown in Equation (5). Since Si-wafer polishing generally requires hydrolysis of the Si surface, an alkaline pH is preferred for the slurry.

(3) $${{(NH}_{4})}_{3}{PO}_{4}\to 3{NH}_{4}^{+}+{PO}_{4}^{3-}$$

(4) $${NH}_{4}^{+}\to {NH}_{3}+H^{+}$$

(5) $$NH_{4}OH\to {NH}_{4}^{+}+{OH}^{-}$$

Despite the increase in TAP concentration from 0.0024 to 0.0244 wt%, the secondary abrasive diameter (120 nm), zeta potential of colloidal silica abrasives (−52 mV), and slurry viscosity (~0.98 cP) remained constant. However, due to the increased NH_4_OH added as a titrant, the NH_3_ odor level significantly increased from 196 to 432 ppm, as shown in Figure 3a. These results suggest that increasing the TAP concentration does not affect the repulsive electrostatic force between the colloidal silica abrasives and the hydrolyzed Si surface, suggesting that the polishing enhancement by TAP is not governed by mechanical effects. When CMP was performed using the slurry with varying TAP concentrations, the Si polishing rate increased considerably from 0.4 to 12.5 nm/min, and the surface roughness of the polished wafer also noticeably increased from 0.114 to 0.254 nm, as shown in Figure 3b. To investigate the cause of polishing rate enhancement, the OH^−^ mol concentration in the slurry was examined. As the TAP concentration increased, the amount of NH_4_OH required to maintain pH 9.7 increased from 0.216 to 0.513 g. Generally, the increase in OH^−^ mol concentration in the Si-wafer CMP slurry promoted hydrolysis reactions between the positively charged Si atoms (i.e., Si dangling bonds) on the Si surface and OH^−^ ions, resulting in the formation of soluble Si(OH)_4_ and three electrons, as described in Equation (6):

(6) $${Si}^{+}+4{OH}^{-}\to {Si\left(OH\right)}_{4}+3e^{-}$$

The addition of TAP in the slurry produces the dissociation of TAP into 3NH_4_^+^ and PO_4_^3−^, as shown in Equation (3), and then 3NH_4_^+^ is mainly dissociated with NH_3_ and H^+^, as shown in Equation (4). Particularly, although PO_4_^3−^ reacted with H_2_O in the slurry is able to be dissociated with HPO_4_^2−^ and OH^−^, HPO_4_^2−^ is simultaneously dissociated with PO_4_^3−^ and H^+^. As a result, PO_4_^3−^ reacted with H_2_O in the slurry would not considerably increase the pH of the slurry. Thus, the addition of TAP in the slurry dominantly results in the dissociation of 3NH_4_^+^ into NH_3_ and H^+^, so that its pH decreases from 7.92 to 7.11 when the TAP concentration changes from 0.0024 to 0.0244 wt%, as shown in Figure 3a. To titrate the pH of the Si-wafer CMP slurry toward 9.7, the amount of the titrant NH_4_OH increased from 0.216 to 0.513 g. Note that the titrant NH_4_OH in the slurry is dissociated with NH_4_^+^ and OH^−^. This dissociation rate of NH_4_OH into NH_4_^+^ and OH^−^ is dependent of the slurry pH, i.e., a lower pH (i.e., pH shift into acid) leads to a lower dissociation rate of NH_4_OH into NH_4_^+^ and OH^−^. For example, the dissociation rate of NH_4_OH into NH_4_^+^ and OH^−^ at pH 7.92, caused by adding 0.0024 wt% TAP, would be estimated as 1.04 × 10^−7^ while at pH 7.11, caused by adding 0.0244 wt% TAP, would be estimated as 1.61 × 10^−8^, as shown in Table S2. Thus, to titrate the slurry pH including TAP toward 9.7, the amount of the titrant NH_4_OH should be increased with the TAP concentration in the slurry, depending on the slurry pH, as shown in Figure 3a. As well, the remained un-dissociated titrant NH_4_OH increased with the TAP concentration in the slurry, i.e., a higher TAP concentration in the slurry would lead to a higher amount of the remained un-dissociated titrant NH_4_OH in the slurry. This remaining un-dissociated titrant NH_4_OH would also enhance a hydrolysis reaction in the Si-wafer surface during CMP, since it presented between the colloidal silica abrasives, and the CMP pad would be dissociated into NH_4_^+^ and OH^−^ during CMP. Note that the necessary dissociation energy of NH_4_OH could be supplied by the friction energy between colloidal silica abrasives and the CMP pad during CMP. Thus, although the pH of all slurries was titrated into 9.7 so that the OH^−^ concentration were same for all slurries; the concentration of the remained un-dissociated NH_4_OH increased with the TAP concentration in the slurry so that the hydrolysis reaction degree, via un-dissociated NH_4_OH into the dissociation of NH_4_^+^ and OH^−^ during CMP, increased with the TAP concentration in the slurry. As a result, the Si-wafer polishing rate significantly increases with the TAP concentration in the slurry, as shown in Figure 3b.

**Figure 4:**
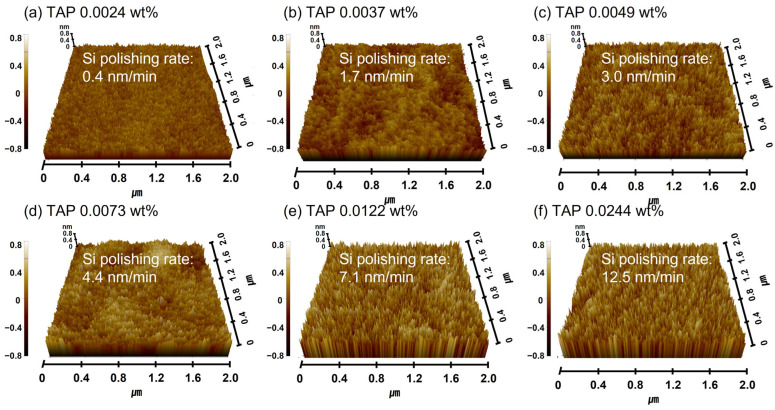
Dependency of surface roughness on TAP concentration based on AFM analysis. AFM surface topography images of Si-wafers after CMP using slurries with different TAP concentrations. (**a**) 0.0024 wt%, (**b**) 0.0037 wt%, (**c**) 0.0049 wt%, (**d**) 0.0073 wt%, (**e**) 0.0122 wt%, and (**f**) 0.0244 wt%.

This will be further confirmed by demonstrating that the Si(OH)_4_ chemical bonding intensity on the polished Si surface increases with the TAP concentration. Meanwhile, the abrasive drag force remained constant at ~9.2 × 10^−10^ N, even as the TAP concentration increased. As the slurry viscosity remained constant, the abrasive drag force was also unaffected, suggesting that the polishing rate enhancement was not governed by viscosity-induced mechanical effects. Additionally, the increase in the Si polishing rate from 0.4 to 12.5 nm/min with TAP concentration also caused the surface roughness of the polished Si-wafer to increase considerably from 0.114 to 0.254 nm, as shown in Figure 3b and Figure 4. This is attributed to the thickening of the chemically formed soluble Si(OH)_4_ layer due to higher TAP concentrations, which increases the effective contact area between the 100 nm diameter colloidal silica abrasives and the Si-wafer during polishing, thereby elevating both the polishing rate and surface roughness.

### 3.2. Acceleration Mechanism of Hydrolysis Reaction on Si Surface During CMP Using Hydrolysis Reaction Accelerator (i.e., TAP) Concentration in the CMP Slurry

**Figure 5:**
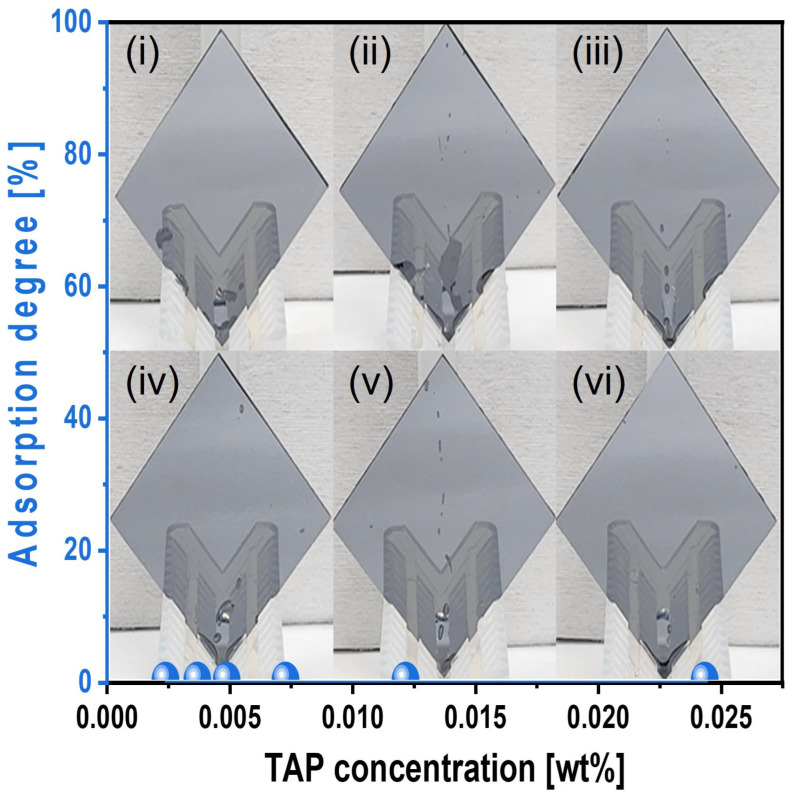
Slurry adsorption degree on the Si-wafer surface, depending on the TAP concentration of the Si-wafer CMP slurry. The Si-wafers were cut into 4 cm × 4 cm for evaluating the adsorption of the slurries depending on the TAP concentration. The cut Si-wafers were dipped into the slurries heated up to 45 °C for 1 min, and then the Si-wafers were vertically loaded for 1 min. No all slurries were adsorbed on the Si-wafer surface. Note that the subfigures of the photographs (**i**–**vi**) of the vertically loaded Si-wafer are the reflected images of the wafer holders.

To elucidate the mechanism by which an increased TAP concentration in the Si-wafer CMP slurry enhances the degree of hydrolysis (i.e., Si(OH)_4_ formation) on the Si surface during CMP, thus increasing the polishing rate, we analyzed the chemical-dominant characteristics of the slurry. First, the adsorption degree of the slurry on the Si-wafer surface was evaluated as a function of TAP concentration. To simulate actual CMP conditions, the TAP-containing Si-wafer CMP slurry was heated to 45 °C, and a Si-wafer was immersed in the heated slurry for 1 min. After removal, the wafer was vertically exposed to ambient air for 1 min, and the adsorption degree was measured on the Si surface, as shown in Figure 5. Despite the increase in TAP concentration from 0.0024 to 0.0244 wt%, the adsorption degree on the Si-wafer surface consistently remained at 0%, indicating that the slurry exhibited hydrophobic characteristics toward the Si surface. This result suggests that even with higher TAP concentrations, no additional slurry adsorption occurs on the Si surface, and thus, TAP does not enhance the polishing rate through increased adsorption. Note that a higher adsorption degree of the Si-wafer CMP slurry generally leads to a higher Si polishing rate [41,42].

**Figure 6:**
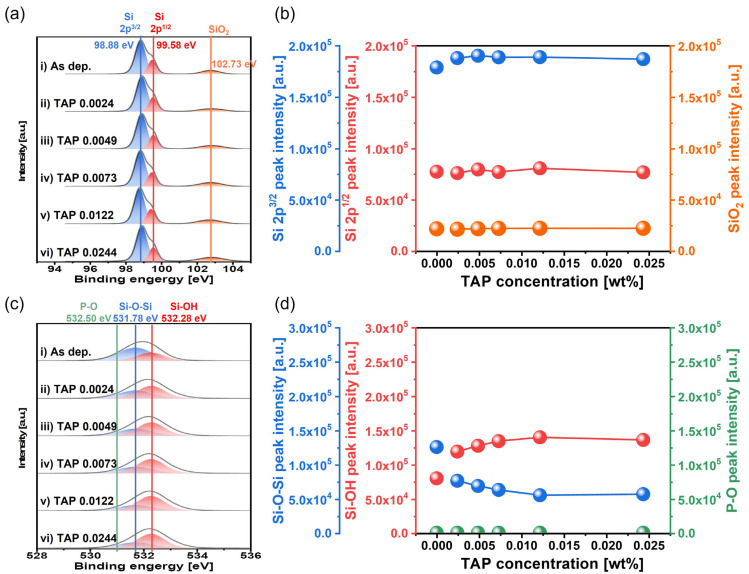
Dependency of surface chemical compositions on TAP concentration based on XPS analysis. (**a**) Si 2p spectra showing the chemical states of elemental Si 2p^3/2^, Si 2p^1/2^, and SiO_2_, (**b**) variation in the peak intensities of Si 2p^3/2^, Si 2p^1/2^, and SiO_2_ with TAP concentration, (**c**) O 1s spectra showing the chemical bonding states of Si-O-Si, Si-OH, and P-O, and (**d**) variation in the peak intensities of Si-O-Si, Si-OH, and P-O as a function of TAP concentration.

To confirm that TAP enhances chemically dominant polishing behavior via hydrolysis, the chemical composition of the polished Si surface was analyzed using X-ray photoelectron spectroscopy (XPS) as a function of TAP concentration, as shown in Figure 6. From the Si 2p spectra, peaks corresponding to Si 2p^3/2^, Si 2p^1/2^, and SiO_2_ were observed at binding energies of 98.88, 99.58, and 102.73 eV, respectively, as shown in Figure 6a. The relative XPS peak intensities of Si 2p^3/2^, Si 2p^1/2^, and SiO_2_ remained unchanged at approximately 18 × 10^4^, 7.5 × 10^4^, and 2.5 × 10^4^ a.u., respectively, regardless of TAP concentration, as shown in Figure 6a,b. In contrast, notable changes were observed in the O 1s spectra. Peaks corresponding to Si-O-Si and Si-OH bonds were located at binding energies of 531.78 and 532.28 eV, respectively, as shown in Figure 6c. As the TAP concentration increased from 0.0024 to 0.0244 wt%, the relative XPS peak intensity of Si-O-Si decreased remarkably from 12 × 10^4^ to 6 × 10^4^ a.u., while that of Si-OH increased significantly from 8 × 10^4^ to 14 × 10^4^ a.u., as shown in Figure 6c,d. This indicates that the relative intensity of the native oxide (Si-O-Si) decreases, while the intensity of soluble Si-OH bonds formed via the hydrolysis reaction increases with a higher TAP concentration. Additionally, the XPS peak of the P-O bond is found at 532.50 eV in binding energy [43]. No XPS peaks of the P-O bond were presented for the polished Si-wafer using the slurries including 0.0024~0.0244 wt% TAP, as shown in Figure 6c,d. These results indicate that the TAP in the slurry does not chemically interact directly with the Si-wafer surface during Si-wafer polishing. Although TAP does not directly adsorb onto the Si surface, its presence in the slurry shifts the pH toward acidity. To maintain the target pH of 9.7, more NH_4_OH must be added, which dissociates to generate additional un-dissociated NH_4_OH in the slurry. These un-dissociated NH_4_OH dissociated with NH_4_^+^ and OH^−^ during CMP and then OH^−^ chemically reacted with the positively charged Si surface, promoting hydrolysis and increasing the formation of the soluble Si(OH)_4_ layer. The dissociation of (NH_4_)_3_PO_4_ into 3NH_4_^+^ and PO_4_^3−^ is an endothermic reaction rather than an exothermic reaction [44,45]. It was confirmed that the temperature of the CMP pad was not varied, although the TAP concentration in the slurry was increased, being constant 20 °C, indicating that the increase of the Si-wafer polishing rate when the TAP concentration in the slurry increased was not associated with a temperature change related to the addition of TAP in the slurry. In addition, the XPS peak of P-O bonds for the polished Si-wafer surface were independent of the TAP concentration in the Si-wafer CMP slurry, as shown in Figure 6c,d. This result implies that the dissociated PO_4_^3−^ from TAP in the slurry does not lead to a hydrolysis reaction on the polished Si-wafer surface to enhance the Si-wafer polishing rate. However, the XPS peak of Si-OH increased evidently with the TAP concentration in the slurry, as shown in Figure 6d, and the addition of TAP in the slurry significantly promoted the hydrolysis reaction degree [i.e., soluble Si(OH)_4_ formation on the Si-wafer surface] during CMP. This promotion of the soluble Si(OH)_4_ formation on the Si-wafer surface caused by adding TAP in the slurry would have originated from un-dissociated titrant NH_4_OH in the slurry. Since the amount of un-dissociated titrant NH_4_OH in the slurry remarkably increased with the TAP concentration, the Si-wafer polishing rate increased significantly with the TAP concentration in the slurry. Thus, OH^−^ ions play the main role in the hydrolysis reaction, while TAP only indirectly promotes this by providing acidity that leads to the addition of more NH_4_OH and thus more OH^−^ ions. In addition, other acidic substances such as monoammonium phosphate (MAP) and diammonium phosphate (DAP) also can promote the hydrolysis reaction in a similar manner as TAP, as shown in Table S3.

Therefore, the observed increase in the polishing rate and surface roughness with an increasing TAP concentration is due to the enhanced hydrolysis reaction: higher OH^−^ mol concentrations in the slurry react with the positively charged Si atoms to produce more Si(OH)_4_, which is then removed by colloidal silica abrasives via chemically dominant polishing, since the Si polishing rate was mainly determined by the TAP concentration of the Si-wafer CMP slurry.

### 3.3. Dependencies of Si Polishing Rate and Polished Si Surface Roughness Increase on Abrasive Drag Force Accelerator (i.e., HEC) Concentration in the CMP Slurry

**Figure 7:**
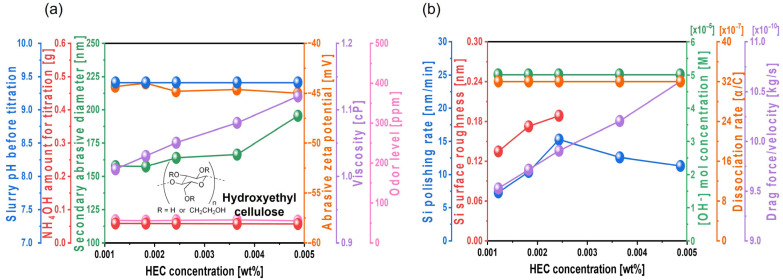
Dependency of Si-wafer CMP slurry properties and polishing performance on HEC concentration. (**a**) Slurry properties, including slurry pH before titration, NH_4_OH consumption for pH adjustment, secondary abrasive diameter, abrasive zeta potential, viscosity, and odor level, and (**b**) Si-wafer CMP performance, including polishing rate, surface roughness, hydroxide ion concentration, dissociation rate of hydroxide ion, and drag force.

To enhance the silicon polishing rate during CMP, TAP was initially mixed into the Si-wafer CMP slurry to increase the hydrolysis reaction degree between the positively charged Si surface and OH^−^ ions in the slurry. Although increasing the TAP concentration can further improve the polishing rate, it requires additional NH_4_OH to maintain the slurry pH at 9.7, thereby significantly increasing NH_3_ odor levels, which raises environmental concerns. As an alternative approach, hydroxyethyl cellulose (HEC), a nonionic polymer, was introduced to increase slurry viscosity and enhance abrasive drag force without increasing NH_3_ odor. Even as the HEC concentration increased from 0.0012 to 0.0049 wt%, the amount of NH_4_OH required to maintain a slurry pH of 9.7 remained constant at ~0.056 g, indicating that HEC does not affect slurry pH directly, as shown in Figure 7a. With increasing HEC concentration from 0.0012 to 0.0037 wt%, the secondary abrasive diameter of the slurry increased slightly from 157.4 to 166.1 nm. However, at 0.0049 wt% HEC, the secondary diameter increased exponentially to 195.3 nm. This result suggests that increased HEC concentration leads to HEC adsorption onto the colloidal silica surface, promoting bridging interactions between HEC and thereby increasing the secondary abrasive diameter. Note that the secondary abrasives in the Si-wafer CMP slurry means the agglomerated colloidal silica abrasives in the Si-wafer CMP slurry, which principally determines the Si-wafer polishing rate. HEC is a non-ionic polymer having hydroxyl functional groups. The hydroxyl functional groups of HEC are readily adsorbed on the surface of colloidal silica abrasives through a condensation process between Si-O-H on the colloidal silica abrasive surface and hydroxyl functional groups (i.e., OHs) of HEC, generating colloidal silica abrasives adsorbed by HEC in the slurry and H_2_O as a by-product [46]. Thus, the addition of HEC in the slurry agglomerated colloidal silica abrasives, since the HEC adsorbed the surface of one or two colloidal silica abrasives, increasing the diameter of the secondary abrasives when the HEC concentration increased. Additionally, as the HEC concentration increased from 0.0012 to 0.0049 wt%, the zeta potential of colloidal silica abrasives remained stable at approximately −45 mV, while the slurry viscosity increased notably from 1.01 to 1.12 cP. The NH_3_ odor level remained constant at ~55 ppm, since the NH_4_OH titrant amount did not change, as shown in Figure 7a.

Furthermore, the Si polishing rate increased significantly from 7.2 to 15.2 nm/min as the HEC concentration increased from 0.0012 to 0.0024 wt%. However, beyond 0.0024 wt%, the polishing rate decreased to 11.2 nm/min. In parallel, the surface roughness of the polished wafer increased considerably from 0.134 to 0.188 nm within the 0.0012 to 0.0024 wt% HEC range. Accurate surface roughness measurement was difficult at HEC concentrations above 0.0024 wt%, as shown in Figure 7b. Despite the increased HEC concentration, the OH^−^ mol concentration in the slurry remained nearly constant at ~5.01 × 10^−5^ M, and the dissociation rate of NH_4_OH into NH_4_^+^ and OH^−^ was also maintained at 32.2 × 10^−7^, indicating that HEC does not influence OH^−^ levels. Thus, the increase in HEC concentration does not contribute to Si(OH)_4_ formation driven by the hydrolysis reaction. These findings confirm that HEC has no effect on the chemically induced polishing rate. However, with an increasing HEC concentration from 0.0012 to 0.0049 wt%, the slurry viscosity increased from 1.01 to 1.12 cP, and the abrasive drag force increased from 9.52 × 10^−10^ to 1.06 × 10^−9^ N. This abrasive drag force accelerator increases the contact probability between the Si surface and colloidal silica abrasives, thereby increasing mechanically dominant Si polishing. Therefore, HEC acts as an abrasive drag force accelerator in the Si-wafer CMP slurry. As the HEC concentration increases, the magnitude of abrasive drag force accelerator also increases, leading to a higher mechanically induced polishing rate. However, while the polishing rate increases linearly and notably with HEC concentrations from 0 to 0.0024 wt%, it decreases beyond this concentration, as shown in Figure 7b. This indicates a dual effect of HEC: further increases in its concentration may hinder polishing, likely due to excessive viscosity or abrasive agglomeration by bridging between abrasives and forming a hindrance layer on the native oxide layer of the Si surface.

### 3.4. Polymeric Hindrance Layer Depending on HEC Concentration in the Si-Wafer CMP Slurry

**Figure 8:**
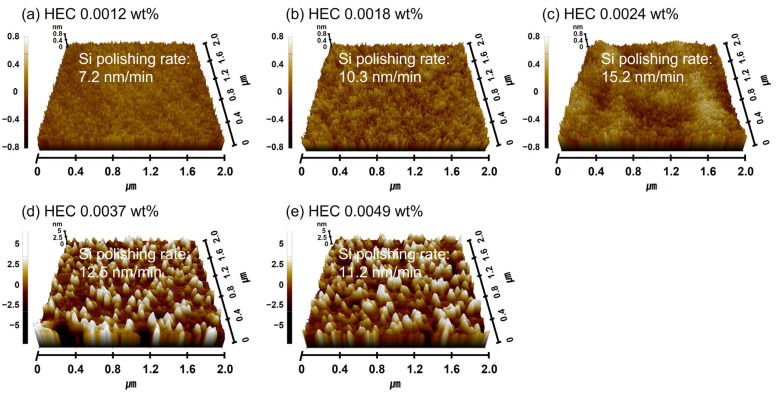
Dependency of surface roughness on HEC concentration based on AFM analysis. AFM surface topography images of Si-wafers after CMP using slurries with different HEC concentrations. (**a**) 0.0012 wt%, (**b**) 0.0018 wt%, (**c**) 0.0024 wt%, (**d**) 0.0037 wt%, and (**e**) 0.0049 wt%.

As shown in Figure 7a, the increase in HEC concentration in the Si-wafer CMP slurry led to enhanced adsorption of HEC onto the colloidal silica abrasive surface, resulting in an increase in the secondary abrasive diameter. However, due to the nonionic nature of HEC, the zeta potential of the abrasives remained unchanged even as the HEC concentration increased. The adsorption of HEC onto colloidal silica abrasive surfaces is attributed to a condensation reaction between the hydroxyl functional groups of HEC and the SiO_2_ surface of the colloidal silica abrasives. In addition to binding with colloidal silica abrasives, HEC can also adsorb onto the native oxide layer of the Si surface via condensation reactions, forming a polymeric hindrance layer. The condensation reaction happened between the hydroxyl functional groups (i.e., OH) of HEC and the Si-OH on the native oxide of the Si-wafer surface, producing the HEC hindrance layers on the Si-wafer surface, as a similar to the condensation reaction between HEC and colloidal silica abrasives [46,47]. As the HEC concentration in the slurry increases, the thickness of this hindrance layer also increases, which, in turn, interferes with the contact between colloidal silica abrasives and the Si surface, ultimately reducing the Si polishing rate. This effect is clearly reflected in the measured surface roughness. As the HEC concentration increased from 0.0012 to 0.0024 wt%, the surface roughness of the polished Si surface increased sharply from 0.134 to 0.188 nm, as shown in Figure 8a–c. When the HEC concentration exceeded 0.0024 wt%, the surface roughness could not be accurately measured due to excessive coating of HEC on the abrasives, which hindered AFM measurement, as shown in Figure 8d,e. To estimate the surface roughness of the polished Si-wafer, the adsorbed HEC hindrance layer should be removed off through the mechanical polishing between colloidal silica abrasives and the HEC hindrance layer; as a result, colloidal silica abrasives can conduct the mechanical polishing between colloidal silica abrasives and the soluble Si(OH)_4_ on the Si-wafer surface. However, if the adsorbed HEC hindrance layer is too thick to remove off the adsorbed HEC hindrance layer during the mechanical polishing between colloidal silica abrasives and the HEC hindrance layer, the colloidal silica abrasives coated by HEC will rather be adsorbed on the Si-wafer surface. Thus, the tips of AFM could not touch directly the polished Si-wafer surface, and it is not possible to measure the surface roughness of the polished Si-wafer surface.

**Figure 9:**
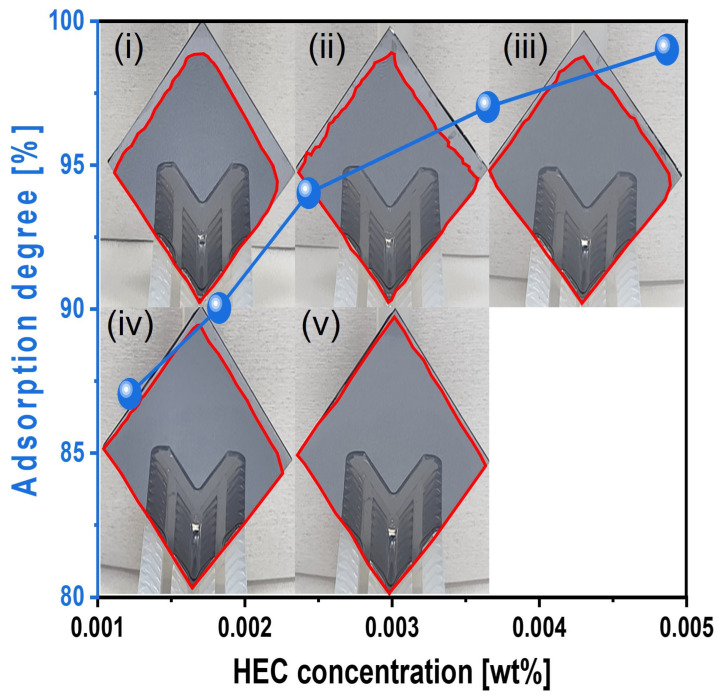
Slurry adsorption degree on the Si-wafer surface depending on the HEC concentration of the Si-wafer CMP slurries. Note that the subfigures of the photographs (**i**–**v**) of the vertically loaded Si-wafer are the reflected images of the wafer holders. The closed red color line on the Si-wafer surface corresponds to the slurry adsorption area (i.e., degree).

The adsorption degree of the slurry onto the Si surface was also evaluated under simulated CMP conditions. Si-wafers were immersed in the slurry heated to 45 °C for 1 min, removed, and vertically exposed to air for 1 min before measurement. The adsorption degree steadily increased from 87 to 99% as the HEC concentration increased from 0.0012 to 0.0049 wt%, as shown in Figure 9. This result confirms that HEC forms a hindrance layer on the native oxide layer of the Si surface, impeding contact with abrasives and reducing the polishing rate.

**Figure 10:**
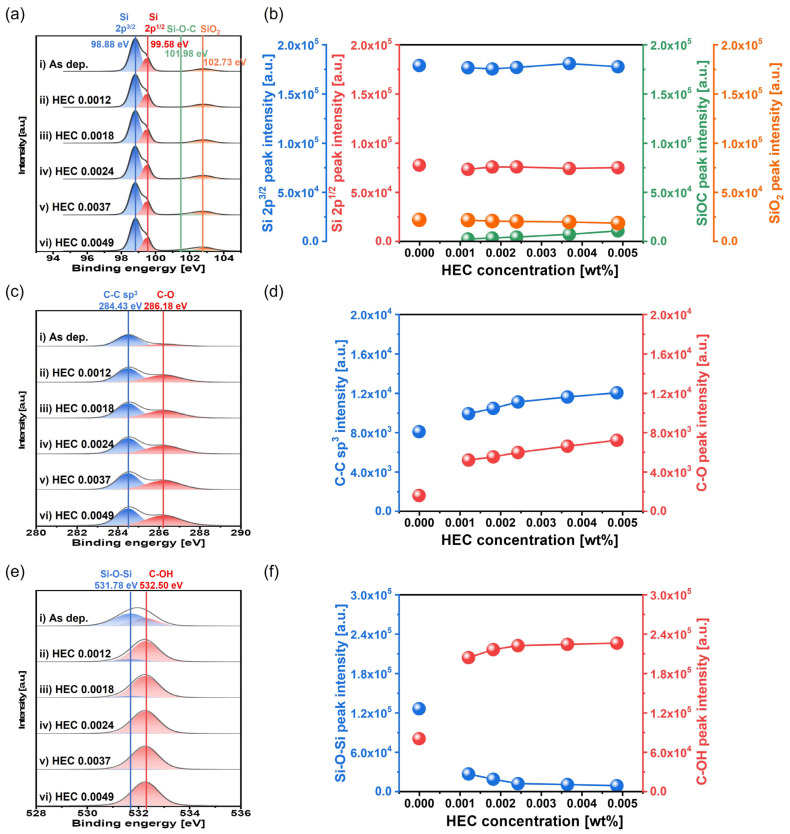
Dependency of surface chemical compositions on HEC concentration based on XPS analysis. (**a**) Si 2p spectra showing the chemical states of elemental Si 2p^3/2^, Si 2p^1/2^, Si-O-C, and SiO_2_, (**b**) variation in the peak intensities of Si 2p^3/2^, Si 2p^1/2^, Si-O-C, and SiO_2_ with HEC concentration, (**c**) C 1s spectra showing C-C and C-O bonding states, (**d**) variation in the peak intensities of C-C and C-O with HEC concentration, (**e**) O 1s spectra showing the chemical bonding states of Si-O-Si and C-OH, and (**f**) variation in the peak intensities of Si-O-Si and C-OH as a function of HEC concentration.

To further verify the mechanically dominant polishing behavior caused by HEC, the chemical composition of the polished Si surface was analyzed via XPS, as shown in Figure 10. In the Si 2p region, peaks corresponding to Si 2p^3/2^, Si 2p^1/2^, Si-O-C, and SiO_2_ were observed at 98.88, 99.58, 101.98, and 102.73 eV, respectively, as shown in Figure 10a. As shown in Figure 10a,b, the relative peak intensities of Si 2p^3/2^, Si 2p^1/2^, and SiO_2_ remained constant at 17.5 × 10^4^, 7.5 × 10^4^, and 2.5 × 10^4^ a.u., respectively, across the HEC concentration range. In contrast, the relative intensity of the Si-O-C bond increased notably from 0 to 10,500 a.u., indicating that the hindrance layer formed by HEC adsorption involves Si-O-C bonding at the Si surface. In the C 1s region, peaks corresponding to C-C and C-O bonds were observed at binding energies of 284.43 and 286.18 eV, respectively, as shown in Figure 10c. As the HEC concentration increased from 0 to 0.0049 wt%, the relative XPS peak intensity of the C-C bond increased significantly from 8037 to 12,014 a.u., while that of the C-O bond sharply increased from 1519 to 7163 a.u., as shown in Figure 10c,d. These increases further confirm that the HEC polymer adsorbs onto the Si surface, forming a hindrance layer via condensation bonding with the native oxide. In the O 1s region, peaks corresponding to Si-O-Si and C-OH bonds were observed at 531.78 and 532.50 eV, respectively, as shown in Figure 10e. As the HEC concentration increased from 0 to 0.0049 wt%, the relative XPS peak intensity of Si-O-Si drastically decreased from 125,602 to 7644 a.u., while the intensity of the C-OH bond sharply increased from 79,680 to 225,781 a.u., as shown in Figure 10e,f. These findings indicate that as the HEC concentration increases, the native oxide (Si-O-Si) layer is replaced by a hydroxyl-rich HEC layer formed through condensation reactions, leading to the growth of a hindrance layer.

In summary, the increase in HEC concentration in the Si-wafer CMP slurry promotes the formation of a polymeric hindrance layer on the Si surface through condensation reactions. This layer obstructs the mechanical interaction between the colloidal silica abrasives and the Si surface, thereby reducing the polishing rate and increasing surface roughness when HEC concentration exceeds 0.0024 wt%.

### 3.5. Peak Polishing Rate Mechanism at a Specific HEC Concentration in the Si-Wafer CMP Slurry

HEC in the Si-wafer CMP slurry exhibits two opposing polishing behaviors during the CMP process. As the HEC concentration increases from 0.0012 to 0.0049 wt%, the slurry viscosity increases from 1.01 to 1.12 cP. Consequently, the abrasive drag force increases from 9.52 × 10^−10^ to 1.06 × 10^−9^ N, enhancing the probability of contact between the Si surface and the abrasives and thereby increasing the mechanically dominant Si polishing rate. In contrast, the increased HEC concentration also promotes its adsorption onto the Si surface, which contains a native oxide layer, via condensation reactions. This leads to the formation of a polymeric hindrance layer, which obstructs the interaction between colloidal silica abrasives and the Si surface, thereby reducing the mechanically dominant polishing rate.

Thus, within the Si-wafer CMP slurry, these two competing effects—(1) abrasive drag force accelerator that promotes polishing and (2) formation of a hindrance layer that suppresses polishing—exist in a trade-off relationship. As a result, the maximum Si polishing rate (15.2 nm/min) is achieved at a specific HEC concentration of 0.0024 wt%, where the positive effect of abrasive drag force accelerator and the negative effect of surface shielding are optimally balanced.

### 3.6. Comparison of Si-Wafer Polishing Mechanism Between Hydrolysis Reaction Accelerator (i.e., TAP) and Abrasive Drag Force Enhancement (i.e., HEC)

Table 1 summarizes a comparison of Si polishing rate enhancements and increases in surface roughness in Si-wafer CMP slurries containing either a hydrolysis reaction accelerator (i.e., TAP) or an abrasive drag force accelerator with a hindrance layer (i.e., HEC). When the TAP concentration increased from 0.0024 to 0.0244 wt% in the Si-wafer CMP slurry, the thickness of the chemically formed, soluble Si(OH)_4_ layer also increased due to accelerated hydrolysis reactions. This led to a significant increase in the polishing rate from 0.4 to 12.5 nm/min, while the surface roughness of the polished Si surface increased from 0.114 to 0.254 nm, as shown in Figure 3b. In contrast, when the HEC concentration increased from 0.0012 to 0.0024 wt%, the mechanical Si-wafer polishing rate was enhanced by increased abrasive drag force. Although HEC also adsorbed onto the native oxide layer of the Si surface via condensation reactions, forming a hindrance layer (i.e., Si-O-R) that could inhibit abrasive contact, the abrasive drag force accelerator effect was dominant within this concentration range. The evidence of the condensation reaction (i.e., Si-O-R) between the native oxide layer of the Si surface and HEC is able to be found by sensing the chemical composition of Si-O-C on the polished Si-wafer surface via XPS analysis, since HEC is composed of (OH-C_6_)_n_. Thus, the presence of the XPS peak signal of Si-O-C bond corresponds to the evidence of Si-O-R produced by the condensation reaction between the native oxide layer of the Si surface and HEC. As a result, the polishing rate increased markedly from 7.2 to 15.2 nm/min, while the surface roughness rose sharply from 0.134 to 0.188 nm. Comparing the Si polishing performance of TAP and HEC, it was found that HEC achieves a higher polishing rate (15.2 nm/min) and lower surface roughness (0.188 nm) at a much lower concentration (0.0024 wt%) than TAP. These results indicate that HEC is a more effective Si polishing rate accelerator than TAP, as it provides both high polishing performance and limited roughness degradation at lower concentrations.

### 3.7. Dependency of Si Polishing Rate and Polished Si Surface Roughness Increase on Hydrolysis Reaction Accelerator (i.e., TAP) and Abrasive Drag Force Accelerator (i.e., HEC) in the CMP Slurry

**Figure 11:**
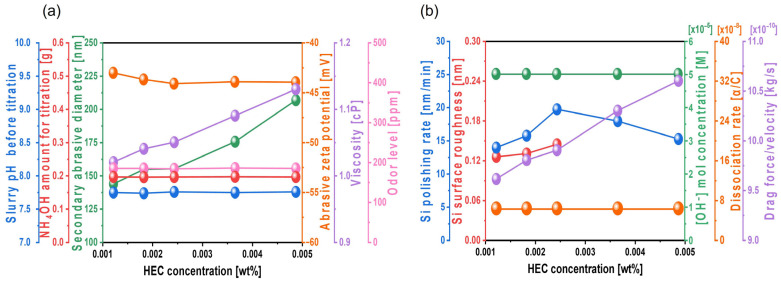
Dependency of Si-wafer CMP slurry properties and polishing performance on HEC concentration with fixed TAP concentration of 0.0037 wt%. (**a**) Slurry properties, including slurry pH before titration, NH_4_OH consumption for pH adjustment, secondary abrasive diameter, abrasive zeta potential, viscosity, and odor level, and (**b**) Si-wafer CMP performance, including polishing rate, surface roughness, hydroxide ion concentration, dissociation rate of hydroxide ion, and drag force.

When TAP is mixed to the Si-wafer CMP slurry to increase the polishing rate, raising its concentration results in increased NH_4_OH usage to maintain the pH at 9.7. This significantly elevates the NH_3_ odor level, leading to environmental concerns, as shown in Figure 3a. In contrast, when HEC is mixed to enhance polishing, its hydroxyl groups form condensation bonds with the native oxide layer on the Si surface, leading to the formation of a hindrance layer that reduces mechanical polishing rate. Additionally, in the HEC-only slurry, excessive adsorption of HEC-coated colloidal silica abrasives made surface roughness unmeasurable by AFM. To optimize these issues and further increase the polishing rate, a composite Si-wafer CMP slurry was developed by combining 0.0037 wt% TAP with varying concentrations of HEC. The slurry pH was maintained at 9.7, and the solid loading of colloidal silica abrasives was fixed at 0.22 wt%. As shown in Figure 11a, even with HEC concentrations ranging from 0.0012 to 0.0049 wt%, the amount of NH_4_OH required for pH adjustment remained constant at ~0.195 g, indicating that HEC does not influence the slurry pH. The secondary abrasive diameter increased slightly from 143.6 to 154.9 nm as the HEC concentration increased to 0.0024 wt% and then rose exponentially to 206.7 nm at 0.0049 wt%. These trends were consistent with those observed in the HEC-only slurry, suggesting that increased HEC leads to adsorption on colloidal silica surfaces and bridging between HEC chains. The zeta potential of the abrasives remained stable at approximately –43 mV, and the viscosity of the slurry increased from 1.02 to 1.13 cP. Because the NH_4_OH amount remained unchanged, the NH_3_ odor level also remained steady at ~184 ppm, as shown in Figure 11a.

**Figure 12:**
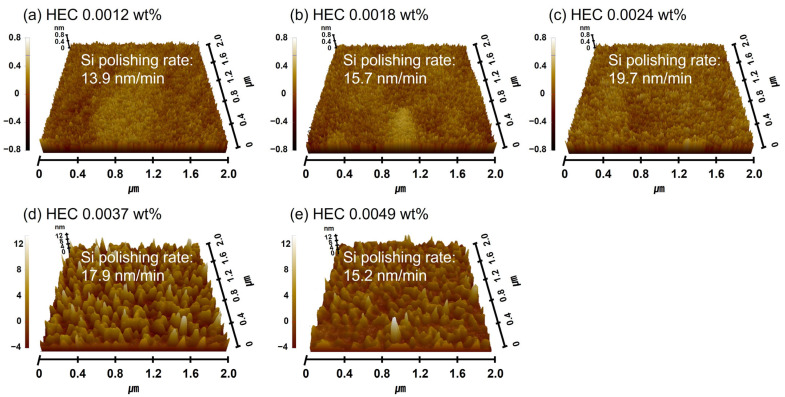
Dependency of surface roughness on HEC concentration with fixed TAP concentration of 0.0037 wt%, based on AFM analysis. AFM surface topography images of Si-wafers after CMP using slurries with different HEC concentrations. (**a**) 0.0012 wt%, (**b**) 0.0018 wt%, (**c**) 0.0024 wt%, (**d**) 0.0037 wt%, and (**e**) 0.0049 wt%.

**Figure 13:**
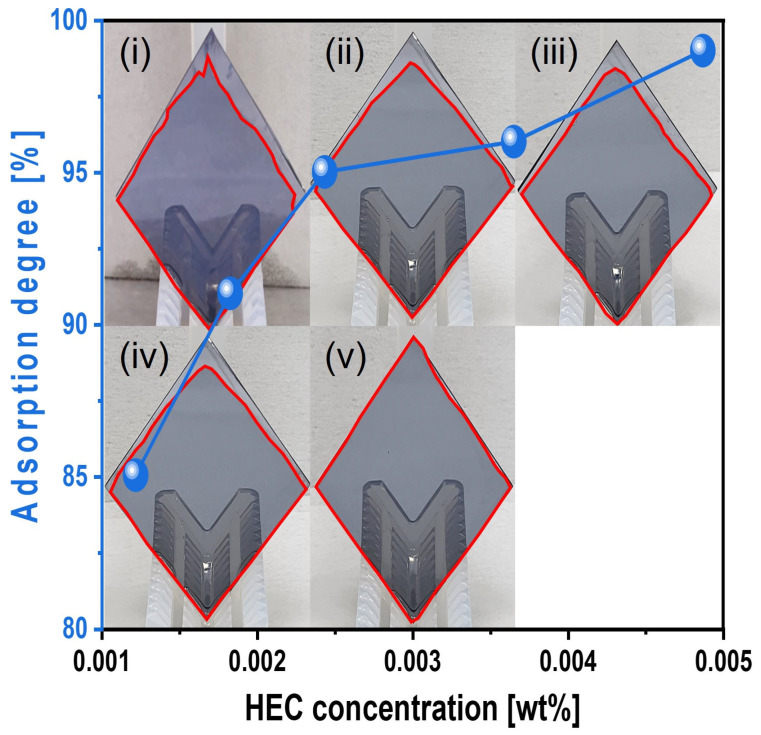
Dependency of surface adsorption behavior on HEC concentration with fixed TAP concentration of 0.0037 wt%. Note that the subfigures of the photographs (**i**–**v**) of the vertically loaded Si-wafer are the reflected images of the wafer holders. The closed red color line on the Si-wafer surface corresponds to the slurry adsorption area (i.e., degree).

Furthermore, with 0.0037 wt% TAP fixed, the polishing rate increased considerably from 13.9 to 19.7 nm/min as the HEC concentration increased from 0.0012 to 0.0024 wt% but then decreased to 15.2 nm/min at higher HEC concentrations, as shown in Figure 11b. The surface roughness also noticeably increased from 0.125 to 0.144 nm in this range. However, as with the HEC-only slurry, surface roughness could not be clearly measured when the HEC concentration exceeded 0.0024 wt% due to severe adsorption of HEC-coated abrasives on the Si surface, as shown in Figure 11b. Even though the HEC concentration increased from 0.0012 to 0.0049 wt%, the OH^−^ mol concentration in the slurry remained constant at ~5.01 × 10^−5^ M, and dissociation rate of NH_4_OH into NH_4_^+^ and OH^−^ also maintained at ~7.7 × 10^−8^. This indicates that HEC does not affect the OH^−^ mol concentration. However, because 0.0037 wt% of TAP was included, more NH_4_OH was consumed to maintain the pH at 9.7 compared to the HEC-only slurry. This likely resulted in a higher degree of hydrolysis and greater formation of the soluble Si(OH)_4_ layer, leading to enhanced chemical-dominant polishing. At the same time, increasing the HEC concentration from 0.0012 to 0.0049 wt% raised the slurry viscosity from 1.02 to 1.13 cP and the abrasive drag force from 9.61 × 10^−10^ to 1.06 × 10^−9^ N. This increased contact probability between the colloidal silica abrasives and the Si surface, improving mechanical-dominant polishing. Thus, HEC acted as an abrasive drag force accelerator chemical in the composite slurry. However, as observed in the HEC-only slurry, the polishing rate increased clearly and gradually up to 0.0024 wt% HEC but decreased beyond this concentration, as shown in Figure 11b. The increase in polishing rate with increasing HEC concentration was also reflected in the surface roughness evaluation. As shown in Figure 12, surface roughness increased sharply from 0.125 to 0.144 nm when the HEC concentration increased from 0.0012 to 0.0024 wt%, as shown in Figure 11b and Figure 12a–c. At concentrations above 0.0024 wt%, surface roughness could not be measured due to excessive adsorption of HEC-coated colloidal silica abrasives, as shown in Figure 12d,e. The adsorption degree of the slurry on the Si surface also increased steadily from 85 to 99% as the HEC concentration increased from 0.0012 to 0.0049 wt%, as shown in Figure 13. This result indicates that HEC adsorbs not only onto the colloidal silica abrasives but also onto the native oxide layer on the Si surface through condensation reactions, forming a hindrance layer that disrupts abrasive–surface contact and reduces the polishing rate.

Notably, when comparing slurries with and without TAP at the same HEC concentrations, the TAP-containing slurry consistently exhibited higher polishing rates, as shown in Figure 7b and Figure 11b. For example, at 0.0024 wt% HEC, the polishing rate and surface roughness of the TAP-containing slurry were 19.7 nm/min and 0.144 nm, respectively, while the HEC-only slurry showed 15.2 nm/min and 0.188 nm. These results suggest that in the HEC-only slurry, the hindrance layer formed by condensation between HEC’s hydroxyl groups and the Si surface reduces the polishing efficiency and increases roughness. In contrast, in the composite slurry, TAP increases NH_4_OH consumption and thereby enhances hydrolysis, forming more soluble Si(OH)_4_. This amplifies the chemical–mechanical synergy of HEC, resulting in higher polishing rates and lower surface roughness than in the HEC-only system. The synergy effect on the Si-wafer polishing rate enhancement from mixing of TAP and HEC can be analyzed by comparison of the Si-wafer polishing rate depending on the HEC concentration between using the mixed CMP slurry of TAP and HEC and calculating the Si-wafer polishing rate with various HEC concentrations plus only TAP 0.0037 wt% (1.7 nm/min). The Si-wafer polishing rate using the mixed TAP and HEC slurry was higher than that calculating the Si-wafer polishing rate just using HEC plus just using TAP 0.0037 wt%, as shown in Figure S1. This a result presents a synergy effect on the Si-wafer polishing rate from the Si-wafer CMP slurry mixed with HEC and TAP.

## 4. Conclusions

For the wafer thinning of HBM and 3D NAND Flash Memory, to increase the Si polishing rate and minimize the polished Si surface roughness in Si-wafer CMP using a soft pad, the Si-wafer CMP slurry was designed with a hydrolysis reaction accelerator (i.e., triammonium phosphate: TAP) and an abrasive drag force accelerator (i.e., Hydroxyethyl Cellulose: HEC). The addition of TAP in the Si-wafer CMP slurry significantly increased the un-dissociated titrant NH_4_OH amount under the slurry pH fixed at pH 9.7. Since the un-dissociated titrant NH_4_OH greatly promoted the hydrolysis reaction degree [(i.e., soluble Si(OH)_4_ formation on the Si-wafer surface)], a higher TAP concentration in the slurry led to a higher the hydrolysis reaction degree. As a result, a higher TAP concentration in the slurry led to a higher Si-wafer polishing rate. This is interpreted as TAP enhancing surface reactivity by converting part of the SiO_2_ layer into Si-OH during the CMP process, but excessive hydrolysis reaction reduces surface uniformity. In addition, XPS analysis showed that as the TAP concentration increased from 0.0024 to 0.0244 wt%, the Si-O-Si bond peak intensity decreased, and the Si-OH bond peak intensity increased. The increase in Si-OH peak intensity indicates that TAP promotes hydrolysis on the SiO_2_ surface, forming Si(OH)_4_ and thereby increasing the Si-film polishing rate. HEC in the Si-wafer CMP slurry was found to influence both the Si polishing rate and surface roughness by increasing the slurry viscosity and controlling the abrasive drag force in the slurry. At low concentrations of HEC (i.e., 0.0012~0.0024 wt%), the slurry viscosity increased moderately, maintaining abrasive dispersibility while enhancing the Si polishing rate. However, when the HEC concentration exceeded 0.0037 wt%, the slurry viscosity increased excessively, and slurry adsorption on the Si surface also increased, forming a hindrance layer that led to a decrease in the Si polishing rate. Furthermore, XPS analysis showed that at high HEC concentrations, C-OH bonds increased, indicating that condensation reactions between hydroxyl groups in the slurry and the Si surface became more pronounced. To achieve a high Si polishing rate (≥10 nm/min) and low surface roughness (≤0.2 nm), the optimal Si-wafer CMP slurry composition was TAP at 0.0037 wt% and HEC below 0.0024 wt%. TAP promotes hydrolysis reaction on the SiO_2_ surface by increasing the OH^−^ mol concentration in the slurry, thereby increasing the Si polishing rate, while HEC modifies the physical properties of the slurry to regulate CMP performance. In addition, the designed hydrolysis reaction accelerator (i.e., TAP), i.e., 12.5 nm/min, presented a remarkably higher Si polishing rate than the conventional hydrolysis reaction accelerator (i.e., EDA), i.e., 2.2 nm/min, although the TAP concentration (i.e., 0.024 wt%) was ten times lower than the EDA concentration (i.e., 0.22 wt%) [26]. Further study on whether not only hydrolysis reaction but also chemical oxidation via a phosphate oxidant from a hydrolysis reaction accelerator (i.e., TAP) co-existed or not would be necessary for understanding the Si polishing mechanism in detail.

## Figures and Tables

**Table 1:** Comparison of Si polishing rate enhancement and surface roughness degradation between TAP and HEC.

Si Polishing Rate Accelerator	TAP	HEC
Chemical concentration	0.0024 → 0.0244 wt%	0.0012 → 0.0244 wt%
Si polishing rate	0.4 → 12.5 nm/min	7.5 → 15.2 nm/min
Surface roughness	0.114 → 0.254 nm	0.134 → 0.188 nm
Surface interaction mechanism	Hydrolysis reaction	Drag force enhancement and hindrance layer
Structure 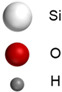	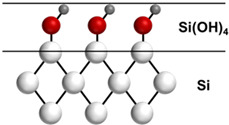	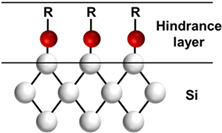

## Data Availability

The raw data supporting the conclusions of this article will be made available by the authors on request.

## Supplementary Materials

The following supporting information can be downloaded at https://www.mdpi.com/article/10.3390/nano15161248/s1: Table S1: Comparative effects of hydrolysis reaction accelerator (i.e., TAP and EDA) on Si polishing rate. Table S2. NH_4_OH dissociation rate depending on slurry pH determined by total addition amount of TAP in the slurry. Table S3. Comparison of Si polishing rate using different phosphate-based hydrolysis reaction accelerators at fixed slurry conditions. Figure S1. Comparison of the Si polishing rate depending on the HEC concentration. (a) Si polishing rate depending on HEC concentration in the Si-wafer CMP slurry mixed with TAP 0.0037 wt% and (b) calculated Si polishing rate depending on HEC concentration in the Si-wafer CMP slurry using just HEC plus the Si polishing rate using just TAP 0.0037 wt% (1.7 nm/min).
