# Supplementary material for: Effects of Hydrolysis Reaction and Abrasive Drag Force Accelerator on Enhancing Si-Wafer Polishing Rate and Improving Si-Wafer Surface Roughness
Source: Nanomaterials (Basel). 2025 Aug 14;15(16):1248. doi: 10.3390/nano15161248 (PMC12388451; doi:10.3390/nano15161248)
Supplement: Supplementary file 1 [file nanomaterials-15-01248-s001.zip › nanomaterials-3753827-supplementary.pdf]

Supplementary Materials

Effects of Hydrolysis Reaction and Abrasive Drag Force Accelerator on Enhancing Si-Wafer Polishing Rate and Improving Si-Wafer Surface Roughness

Min-Uk Jeon [1,†], Pil-Su Kim [2,†], Man-Hyup Han [2], Se-Hui Lee [1], Hye-Min Lee [1], Su-Bin Kim [1], Jin-Hyung Park [3], Kyoo-Chul Cho [4], Jin-Sub Park [1], and Jea-Gun Park [1,2,4,*]

[1] Department of Electronic Engineering, Hanyang University, Seoul 04763, Republic of Korea; mujeon1214@gmail.com (M.-U. J.); eiop77455@gmail.com (S.-H. L.); hm0951911@gmail.com (H.-M. L.); emptybin.kim@gmail.com (S.-B. K.); jinsubpark@hanyang.ac.kr (J.-S. P.)
[2] Department of Nanoscale Semiconductor Engineering, Hanyang University, Seoul 04763, Republic of Korea; psk6208@naver.com (P.-S. K.); aksguq06@naver.com (M.-H. H.)
[3] ENF Technology Co., Ltd., Yongin 17084, Republic of Korea; parkjinhyung@gmail.com (J.-H. P.)
[4] Advanced Semiconductor Materials & Device Development Center, Hanyang University, Seoul 04763, Republic of Korea; kccho12@naver.com (K.-C.C.)
* Correspondence: parkjgl@hanyang.ac.kr
† These authors contributed equally to this work.

Slurry compositions			Si polishing-rate [nm/min]	Reference
Abrasives	Hydrolysis reaction accelerator	pH		
Colloidal silica abrasive 0.22 wt%	triammonium phosphate (TAP) 0.024 wt%	9.7	12.5	This work
Colloidal silica abrasive 0.22 wt%	Ethylenediamine (EDA) 0.22 wt%	10.5	2.2	[26]

Table S1. Comparative effects of hydrolysis reaction accelerator (i.e., TAP and EDA) on Si polishing-rate.

Abrasives	Colloidal silica abrasive 0.22 wt%					
Hydrolysis reaction accelerator [wt%]	0.0024	0.0037	0.0048	0.0073	0.0120	0.0244
Slurry pH before titration	7.92	7.75	7.64	7.59	7.36	7.11
NH_4OH dissociation rate (α/C)	1.04×10^{-7}	7.03×10^{-8}	5.46×10^{-8}	4.87×10^{-8}	2.86×10^{-8}	1.61×10^{-8}

Table S2. NH_4OH dissociation rate depending on slurry pH determined by total addition amount of TAP in the slurry.

Slurry compositions			Si polishing-rate [nm/min]	Reference
Abrasives	Hydrolysis reaction accelerator	pH		
Colloidal silica abrasive 0.22 wt%	monoammonium phosphate (MAP) 0.024 wt%	9.7	6.4	This work
Colloidal silica abrasive 0.22 wt%	diammonium phosphate (DAP) 0.024 wt%	9.7	8.6	This work
Colloidal silica abrasive 0.22 wt%	triammonium phosphate (TAP) 0.024 wt%	9.7	12.5	This work

Table S3. Comparison of Si polishing-rate using different phosphate-based hydrolysis reaction accelerators at fixed slurry conditions.

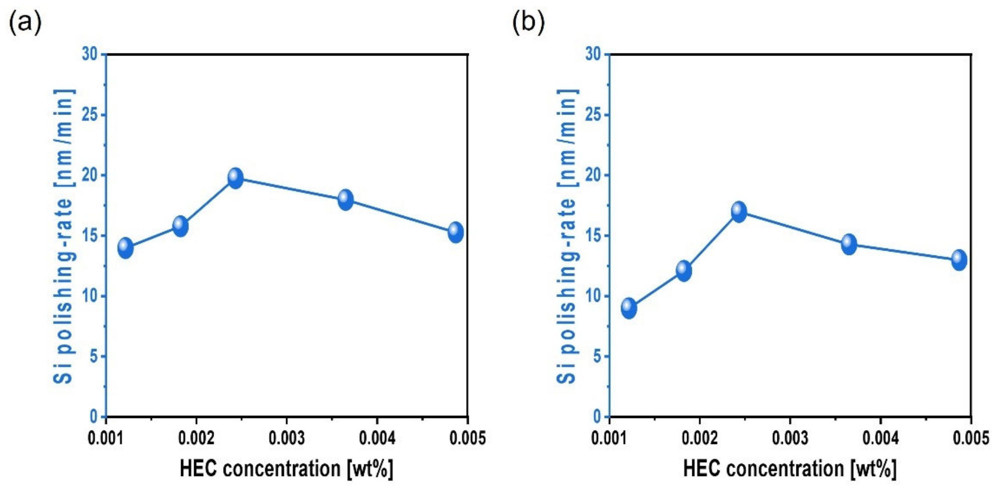

Figure S1. Comparison of the Si polishing-rate depending on the HEC concentration. (a) Si polishing-rate depending on HEC concentration in the Si-wafer CMP slurry mixed with TAP 0.0037 wt%, and (b) calculated Si polishing-rate depending on HEC concentration in the Si-wafer CMP slurry using just HEC plus the Si polishing-rate using just TAP 0.0037 wt% (1.7 nm/min).
